# Survival nomogram for patients with thymic squamous cell carcinoma, based on the SEER database and an external validation cohort

**DOI:** 10.1007/s12672-023-00720-4

**Published:** 2023-06-20

**Authors:** Yelan Guan, Feiqi Xu, Shuai Zheng, Xiaodong Gu, Yan Sun

**Affiliations:** 1 Department of Clinical Trial, Zhejiang Cancer Hospital, Hangzhou Institute of Medicine (HIM), Chinese Academy of Sciences, Hangzhou, 310022 Zhejiang China; 2 Postgraduate training base Alliance of Wenzhou Medical University, Zhejiang Cancer Hospital, Hangzhou, 310022 Zhejiang China; 3 The Second School of Clinical Medical, Zhejiang Chinese Medical University, Hangzhou, 310053 Zhejiang China; 4 The First Affiliated Hospital of Jinan University, Jinan University, Guangdong, 510630 Guangzhou China

**Keywords:** SEER, Nomogram, Thymic squamous cell carcinoma, Survival rate, External validation

## Abstract

**Objective:**

This study aimed to construct a nomogram to effectively predict the 3 years and 5 years overall survival of patients with thymic squamous cell carcinoma (TSCC).

**Method:**

From 2000 to 2019, a total of 355 patients with TSCC were enrolled in our research from the Surveillance, Epidemiology, and End Results (SEER) database and used as the training cohort. 106 patients were included from the Zhejiang Cancer Hospital, for the external validation cohort. A nomogram was constructed based on the risk factors affecting prognosis using a Cox proportional hazards regression model. The discrimination and calibration of the nomogram were evaluated by C-index and curve of calibration. The two cohorts were divided into low-risk and high-risk subgroups based on the median risk score.

**Results:**

Age (*p* = 0.002), stage (*p*** = **0.003), surgery therapy *(p*** < **0.001), and radiotherapy (*p* = 0.030) were the independent prognostic factors for overall survival and were incorporated in the prognostic model. The discrimination of the nomogram revealed a good prognostic accuracy and clinical applicability as indicated by C-index values of 0.696 (95% confidence interval [CI] 0.676–0.716) and 0.717 (95% CI 0.640–0.794) for the training cohort and external validation cohort, respectively. In addition, the two cohorts were divided into a high-risk group and a low-risk group according to the median risk score. Significant differences in overall survival were observed between the high-risk and low-risk groups in the training (*p* < 0.0001) and external validation cohort (*p* < 0.0001).

**Conclusion:**

We developed a nomogram to predict 3- and 5 year survival rate for TSCC. This nomogram provides a convenient and reliable tool for assessing the condition of patients with TSCC and assisting clinicians in making decisions.

**Supplementary Information:**

The online version contains supplementary material available at 10.1007/s12672-023-00720-4.

## Introduction

Thymic carcinomas (TCs) are tumors originating from thymic epithelial cells within the mediastinum, with an incidence ranging from 0.07 to 0.38 per 100,000 individual years [1]. TCs are categorized as high-grade, aggressive cancers with characteristic cellular atypia and infiltrative growth patterns on histological. Among the various pathological types of TCs, thymic squamous cell carcinoma (TSCC) is the most prevalent, accounting for 70–80% of all cases, and most commonly presents as a well-differentiated subtype [2, 3]. Early-stage TCs may be treated with complete thymectomy, which is considered a curative therapy. For advanced TCs, a combination of carboplatin and paclitaxel is often used as the first-line treatment. However, determining the optimal treatment regimen for advanced TCs with regard to efficacy and survival time remains a topic of controversy [4]. As a subtype of TCs, TSCC lacks a standardized treatment approach. Due to the non-specific clinical presentation of early-stage TCs, nearly half of all patients are diagnosed at an advanced stage [5, 6]. According to previous studies, the five-year survival rate for patients with TCs ranges from 30 to 55% [7–11].

The primary focus of current research is to investigate the clinical efficacy of novel drugs, such as immune checkpoint inhibitors and targeted therapies, in treating advanced cases of TCs. However, the prognosis of patients with TCs remains poorly understood due to a scarcity of relevant evidence. Recently, a few studies have utilized the data from Surveillance, Epidemiology, and End Results (SEER) database to identify the prognostic factors affecting the overall survival of patients with TSCC [12, 13]. Nevertheless, the veracity of these findings has not been substantiated using real-world data. Therefore, we have included data from the Zhejiang Cancer Hospital in this manuscript for the purpose of external validation.

## Methods

### Patients and selection criteria

Data was collected from the SEER database by utilizing the National Cancer Institute’s SEER*Stat Database (version 8.4.0.1): Incidence-SEER Research Plus Data, 17 Registries, Nov 2021 Sub (2000–2019), which was made available in April 2022. A total of 423 patients diagnosed with TSCC were identified from the SEER database. Among these cases, fourteen had a survival time of 0 or unknown duration, forty-one were lacking information on SEER stage, four cases had no information on the type of surgery performed, and nine cases were recommended for radiotherapy but had unknown whether it was performed. After excluding these cases, a total of 355 patients were included as training cohort subjects in this research.

We employed the third edition (ICD-O-3) of the primary site codes, which identified the thymus (C37.9), and histological codes, which identified squamous cell carcinoma (8070–8074/3), as our search criteria. The following fields were extracted from the database: demographics, year of diagnosis, marital status at the time of diagnosis, SEER stage, tumor size, distant metastatic sites, information on surgery treatment (yes or no/unknow), surgical procedures performed (no surgery; incomplete resection including local excision, debulking, and partial removal; complete resection including total resection and radical surgery), records of radiotherapy (yes or no/unknow), chemotherapy (yes or no/unknow), and duration of survival in months.

### Construction of the nomogram

In this research, we utilized SPSS version 25 (IBM Inc. Chicago, IL, USA) and R 4.2.1 (http://www.rproject.org) software for the data analysis. Overall survival (OS) was defined as the period from the time of diagnosis to the date of death or the last follow- up time. We employed Cox proportional hazards regression models to evaluate the association between clinical characteristics and OS. P-value, hazard ratios (HRs), and 95% confidence interval (CI) were calculated. Significant variables (*p* < 0.05) were included in multivariate analysis based on the results of univariate analysis. A two-tailed p-value less than 0.05 was considered as statistically significant. The independent risk factors selected based on the results of multivariate analysis were incorporated into the nomogram to predict the likelihood of 3 year and 5 year OS rates in patients with TSCC.

### Discrimination and calibration of the nomogram

The evaluation of the performance of the nomogram included discrimination and calibration. The C-index was used as an indicator of the discriminative ability of the nomogram, which roughly be equivalent to the area under the curve (AUC), with values ranging from 0.5 to 1. The C-index of 0.5 indicates no discrimination ability, while a value of 1 indicates perfect discrimination. Typically, values below 0.6 indicate low differentiation, values between 0.6 and 0.75 indicate moderate differentiation, and a value above 0.75 indicate high differentiation. The calibration curve was used for calibration evaluation. The relationship between the predicted probability and the real probability can be seen intuitively by the graph. If the curve is a straight line with a slope of 1 through the origin, the nomogram has a perfect ability to predict real events. The closer the calibration curve is to the slope of 1, the better the predictive ability of the nomogram.

### Different risk groups stratified by the nomogram

The ‘‘survminer’’ R package was employed to compute the aggregate score for each patient using the nomogram, following which the median score was determined to stratify them into high-risk and low-risk groups. Subsequently, we applied Kaplan–Meier survival analysis and log-rank test to gauge the statistical significance of survival disparities between the two groups. Significance was inferred if *p* < 0.05.

## Results

### Training cohort

**Fig. 1:**
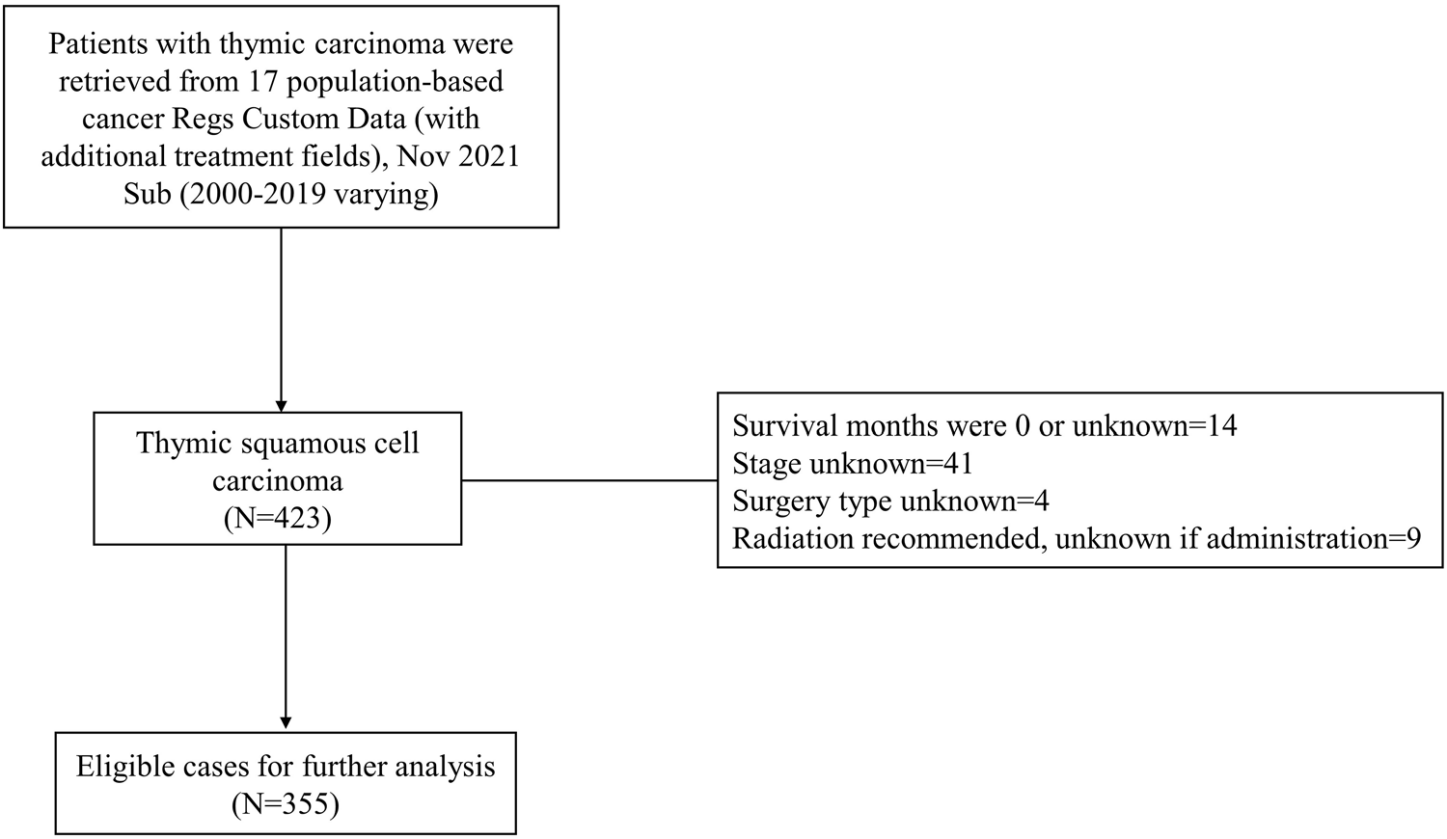
The Flowchart of patient selection for TSCC

The SEER database was queried to retrieve data for 780 patients with thymic carcinoma diagnosed between 2000 and 2019. Out of the total, 355 (45.5%) patients with TSCC were identified and included in the training cohort. Furthermore, 106 patients from Zhejiang Cancer Hospital were selected for the external validation cohort. The inclusion process for the training cohort was presented in Fig. 1.

A total of 461 patients diagnosed with TSCC were included in this research (Table 1). In the training cohort, the median age of diagnosis was 65 years (range, 24–92), with a higher proportion of patients (75.8%) diagnosed in the period 2010–2019 compared to 2000–2010 (24.2%). Among the training cohort, 223 (62.8%) were male and 132 (37.2%) were female. The majority of patients were white ethnicity (66.2%), while Asian or Pacific Islander and patients of other ethnic groups accounted for 20.8% and 13.0%, respectively. The SEER stage at diagnosis was classified as localized in 16.9%, regional in 47.6%, and distant in 35.5% of the cases. Of the known tumor sizes, 5–10 cm (n = 167, 47.3%) was the most common, followed by 0–5 cm (n = 104, 29.3%), and > 10 cm (n = 22, 6.2%). Moreover, 50.0% (177/355) of patients underwent surgery, regardless of the type of surgery. Furthermore, more than half of the patients (n = 198, 55.8%) received radiation therapy and 58.3% of patients received chemotherapy. The most common sites of metastasis were the lungs (n = 41, 11.5%), followed by bone (n = 26, 7.3%) and liver (n = 13, 3.7%).

**Table 1:** Clinical characteristics of patients

	Training cohort (n = 355)	Validation cohort (n = 106)	P value
Mean age of diagnosis (rang)	65 (24–92)	54 (27–74)	< 0.001*
Time of diagnosis			< 0.001**
2000–2010	86 (24.2)	5 (4.7)	
2011–2021	269 (75.8)	101 (95.3)	
2011–2016	147 (54.6)	27 (26.7)	
2017–2021	122 (45.4)	74 (73.3)	
Age of diagnosis, year			< 0.001**
20–39	18 (5.1)	8 (7.5)	
40–59	96 (27.0)	65 (61.3)	
60–79	197 (55.5)	33 (31.1)	
≥ 80	44 (12.4)	0	
Gender			0.545**
Male	223 (62.8)	70 (66.0)	
Female	132 (37.2)	36 (34.0)	
Race			< 0.001**
White	235 (66.2)	0 (0.0)	
Asian or Pacific Islander	74 (20.8)	106 (100.0)	
Others	46 (13.0)	0 (0.0)	
Marital status			< 0.001**
Married	290 (81.7)	106 (100.0)	
Never married	65 (18.3)	0 (0.0)	
SEER stage			< 0.001**
Localized	60 (16.9)	19 (17.9)	
Regional	169 (47.6)	12 (11.3)	
Distant	126 (35.5)	75 (70.8)	
Tumor size (cm)			0.502**
0–5	104 (29.3)	36 (34.0)	
5–10	167 (47.3)	48 (45.3)	
> 10	22 (6.2)	3 (2.8)	
Unknown	62 (17.5)	19 (17.9)	
Surgery			0.131**
Yes	177 (50.0)	44 (41.5)	
None/unknown	178 (50.0)	62 (58.5)	
Radiotherapy			< 0.001**
Yes	198 (55.8)	89 (84.0)	
None/unknown	157 (44.2)	17 (16.0)	
Chemotherapy			< 0.001**
Yes	207 (58.3)	99 (93.4)	
None/unknown	148 (41.7)	7 (6.6)	
Distant metastatic site			
Lung metastasis			< 0.001**
Yes	41 (11.5)	41 (38.7)	
None/unknown	314 (88.5)	65 (61.3)	
Liver metastasis			< 0.001**
Yes	13 (3.7)	27 (25.5)	
None/unknown	342 (96.3)	79 (74.5)	
Bone metastasis			< 0.001**
Yes	26 (7.3)	30 (28.3)	
None/unknown	329 (92.7)	76 (71.7)	

^*^P value calculated by Wilcoxon Mann–Whitney test

^**^P value calculated by Chi-square test

### Validation cohort

A total of 106 patients were included in the validation cohort after satisfying the following inclusion criteria: 1) histopathological confirmation of TSCC; 2) receipt of at least one of the following treatments at our institution: surgical resection (including radical surgical resection and tumor reduction surgery), chemotherapy (including induction chemotherapy, radical chemotherapy, adjuvant chemotherapy, and palliative chemotherapy), or radiotherapy (including radical radiotherapy, adjuvant radiotherapy, and palliative radiotherapy). Patients for whom survival information could not be recorded were excluded from the external cohort. The stage of the disease, which was defined as the patient's pathological or radiographic status at the end of follow-up on April 23, 2023, was categorized into local, regional, and distant. The training cohort had a median follow-up time of 57.0 months (95%CI 46.1–67.9 months), while the validation cohort had a median follow-up time of 58.2 months (95%CI 47.9–68.4 months).

In this study cohort, the median age at diagnosis was 54 years (range, 27–74). The majority of patients (95.3%) were diagnosed between 2011 and 2021, particularly within the last five years. Regarding age of diagnosis, patients between the ages of 40 and 59 (61.3%) were more frequently diagnosed than those in the training cohort. In terms of gender distribution, male patients (n = 70, 66.0%) were nearly twice as prevalent as female patients (n = 36, 34.0%), which was consistent with the training cohort. The most frequently observed tumor size was 5–10 cm (n = 48, 45.3%). Additionally, 44/106 (41.5%) of patients received surgical resection, while 89/106 (84.0%) and 99/106 (93.4%) received radiotherapy and chemotherapy, respectively. Overall, participants in the validation cohort were younger, more diagnosed within the last 5 years, and more actively treated than those in the training cohort. Lung metastasis (n = 41, 38.7%) was the most frequently observed site of metastasis, followed by bone (n = 30, 38.3%) and liver (n = 27, 25.5%).

In the therapeutic modalities documented at our institution, a vast majority of patients were administered chemotherapy. Among them, the preferred treatment regimen was paclitaxel in combination with platinum, which was prescribed to sixty-six patients for induction chemotherapy, adjuvant chemotherapy, and palliative chemotherapy. Docetaxel in combination with platinum was the preferred treatment option for thirteen patients. Other chemotherapy options comprised gemcitabine, vinorelbine and etoposide in combination with platinum-based chemotherapy. Following complete surgical resection in early-stage patients, adjuvant platinum-containing chemotherapy and adjuvant radiotherapy were administered to almost all patients. Unfortunately, a notable fraction of early-stage patients inevitably developed metastases and recurrence despite undergoing radical surgery and adjuvant radiotherapy treatment. Furthermore, none of these patients responded positively to subsequent treatment options.

For patients who exhibited progression during initial treatment with platinum-based chemotherapy, the subsequent treatment regimens were more varied. These included immunotherapy agents, such as immune checkpoint inhibitors, combined with chemotherapy, ICIs combined with anti-angiogenic drugs (such as anlotinib, apatinib, and sunitinib), monotherapy with chemotherapy, as well as participation in clinical trials, such as NCT04469725.

### Independent risk factors in the training cohort

The outcomes of univariate and multivariate analyses in the training cohort were presented in Table 2. The univariate analysis showed significant associations between OS and variables such as age, SEER stage, tumor size, radiotherapy, surgery therapy, liver metastasis, lung metastasis, and bone metastasis (*p* < 0.05). Based on the results of univariate analysis, the above eight variables were selected for multivariate analysis. The analysis revealed that age (≥ 60: HR 1.698, 95%CI 1.211–2.381,* p* = 0.002), SEER stage (regional: HR 1.711, 95%CI 1.014–2.885, *p* = 0.044; distant: HR 2.565, 95%CI 1.470–4.475,* p* = 0.001), radiation therapy (yes: HR 0.698, 95%CI 0.505–0.966, *p* = 0.030) and surgery therapy (yes: HR 0.466, 95%CI 0.326–0.667, *p* < 0.001) were significantly associated with OS.

**Table 2:** Univariate analysis and multivariate analysis in training cohort

	Univariate analysis		Multivariate analysis	
	HR (95% CI)	P value	HR (95% CI)	P value
Time of diagnosis				
2000–2010	Ref			
2011–2021	0.927 (0.672–1.280)	0.647		
Age (year)				
< 60	Ref		Ref	
≥ 60	1.436 (1.044–1.976)	**0.026**	1.698 (1.211–2.381)	**0.002**
Sex				
Male	Ref			
Female	1.125 (0.830–1.524)	0.447		
Race		0.895		
White	Ref			
Asian or Pacific Islander	1.082 (0.751–1.557)	0.673		
Others	1.071 (0.672–1.707)	0.773		
Marital status				
Single/Unknown	Ref			
Married	0.847 (0.558–1.286)	0.435		
SEER stage		** < 0.001**		**0.003**
Localized	Ref		Ref	
Regional	0.301 (0.180–0.812)	< 0.001	1.711 (1.014–2.885)	0.044
Distant	3.323 (1.991–5.546)	< 0.001	2.565 (1.470–4.475)	0.001
Tumor size (cm)		**0.006**		
0–5	Ref			0.063
5–10	1.525 (1.036–2.244)	0.032	1.358 (0.909–2.029)	0.135
> 10	2.837 (1.473–5.460)	0.002	2.603 (1.281–5.291)	0.008
Unknown	1.850 (1.182–2.895)	0.007	1.238 (0.763–2.010)	0.387
Chemotherapy				
None/unknown	Ref			
Yes	1.272 (0.934–1.731)	0.126		
Radiotherapy				
None/Unknown	Ref		Ref	
Yes	0.568 (0.421–0.768)	** < 0.001**	0.698 (0.505–0.966)	**0.030**
Surgery therapy				
No surgery	Ref		Ref	
Yes	0.369 (0.268–0.507)	** < 0.001**	0.466 (0.326–0.667)	** < 0.001**
Distant metastatic site				
Liver metastasis (no vs yes)	3.823 (1.999–7.312)	** < 0.001**	1.192 (0.659–2.155)	0.562
Lung metastasis (no vs yes)	1.784 (1.156–2.753)	**0.009**	1.620 (0.803–3.271)	0.178
Bone metastasis (no vs yes)	2.196 (1.264–3.816)	**0.005**	1.453 (0.882–2.394)	0.143

Bold indicates that the corresponding variable is statistically significant (p < 0.05)

### The Nomogram for predicting OS

**Fig. 2:**
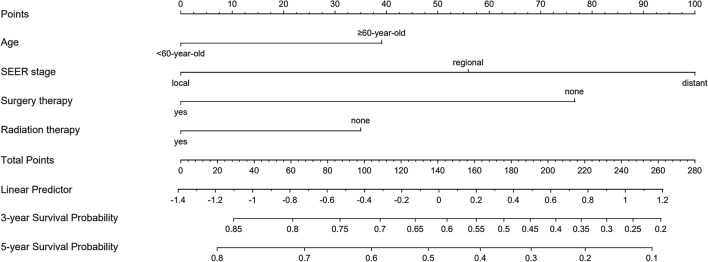
The nomogram of 3 year and 5 year OS prediction of TSCC

A nomogram was created to predict OS based on four independent risk factors: age (< 60 year-old or ≥ 60 year-old), SEER stage (local, regional or distant), radiation therapy (no or yes) and surgery therapy (no or yes) (see Fig. 2). Each independent risk factor corresponds to a specific score by drawing a straight line pointing upwards towards the score axis. The total points reflect the sum of the score of each factor and correspond to the prediction probability of the 3-, and 5 year OS by drawing straight down from the total points axis to the 3-, and 5 year survival axes.

### Discrimination and calibration of the nomogram

**Fig. 3:**
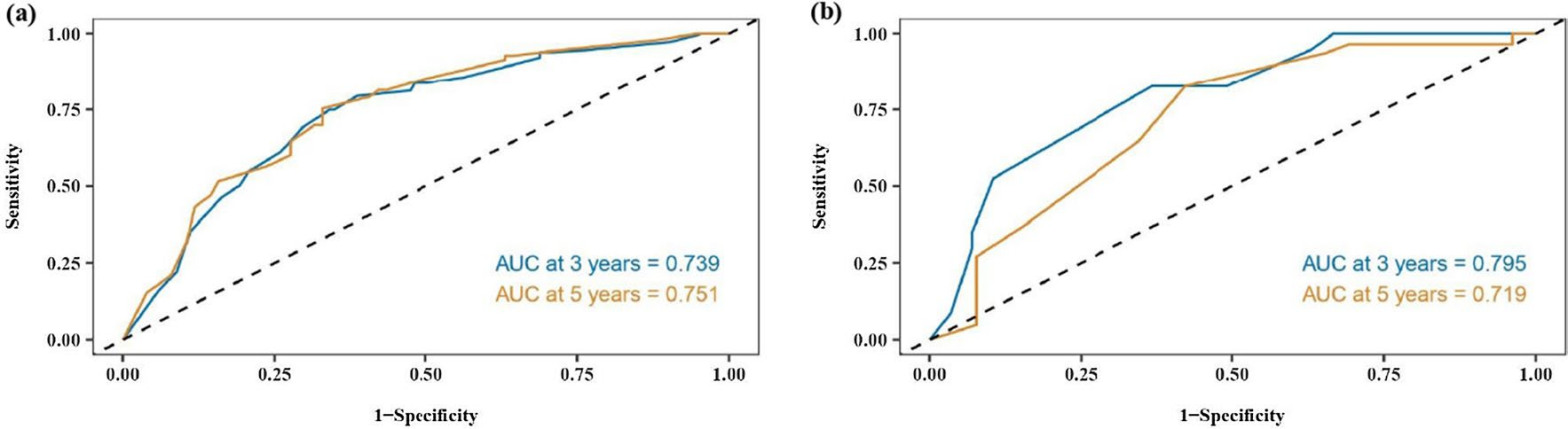
ROC curves and AUCs at 3, and 5 years in the training cohort (**a**) and the external validation cohort (**b**) were used to estimate the prognostic accuracy of the nomogram

**Fig. 4:**
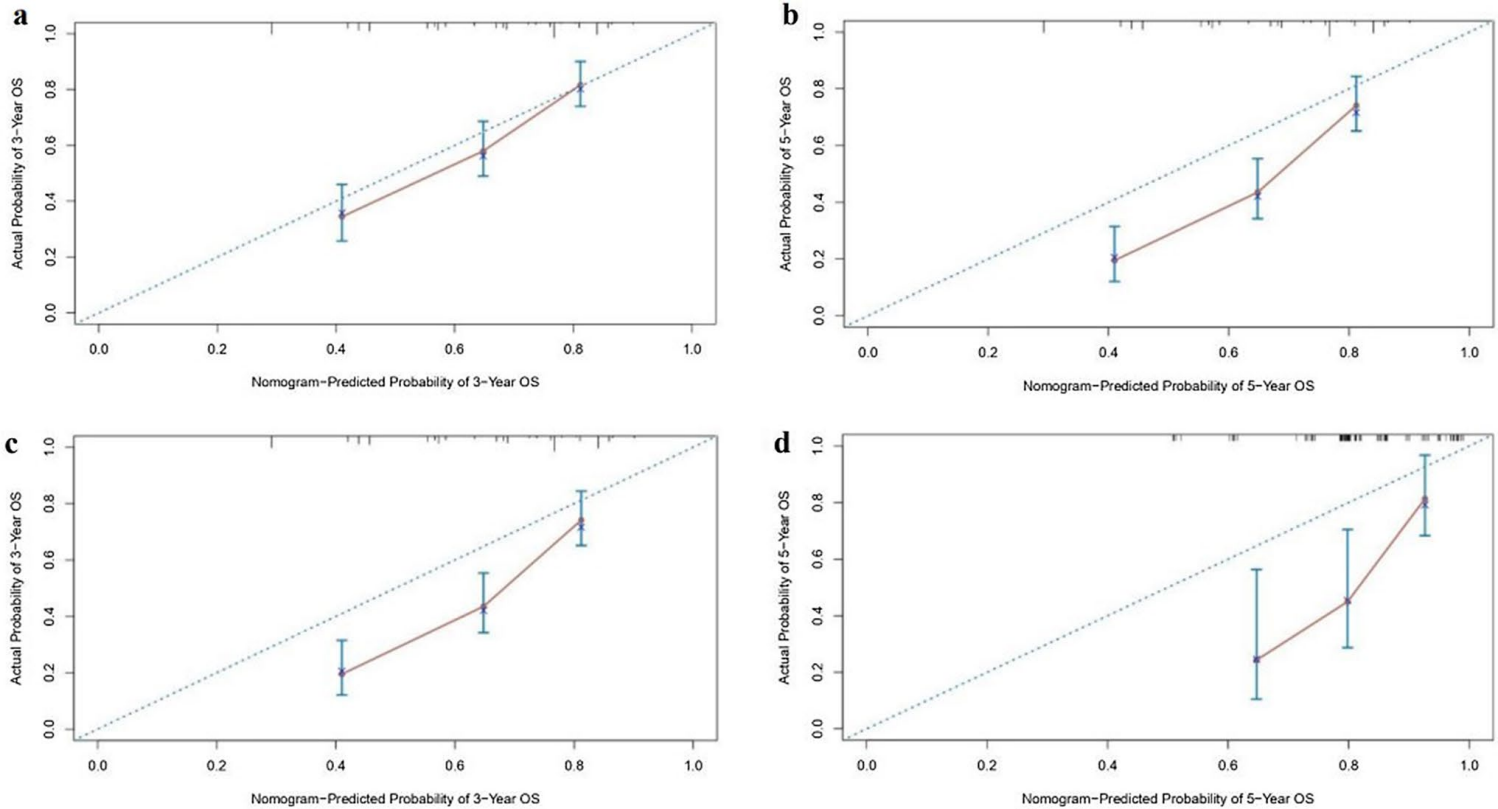
Calibration curves of the training and validation cohort. Calibration curves showing the nomogram-predicted 3- (**a**) and 5 year (**b**) OS probabilities with the actual 3- (**c**), 5 year OS (**d**) in the training cohort and validation cohort, respectively. *OS* overall survival

We assessed the discriminative ability of the nomogram and found that the c-index values were 0.696 (95% CI 0.676–0.716) and 0.717 (95% CI 0.640–0.794) for the training and validation cohorts, respectively. The AUC values for 3 year and 5 year survival were 0.739 and 0.751 for the training cohort, and 0.795 and 0.719 for the validation cohort (Fig. 3). Additionally, calibration curves showed favorable agreement between the predicted and actual observations of the 3- and 5 year OS in both the training and validation cohorts (Fig. 4). Notably, the prediction of the 3 year OS rate was superior. However, the calibration curves for both cohorts indicated that the red line was positioned below the reference line for both 3 year and 5 year survival rates, implying a possible overestimation of the risks. Collectively, our findings support the value of the nomogram in terms of its discriminative ability and clinical utility.

### Ability of the nomogram to stratify patient risk

**Fig. 5:**
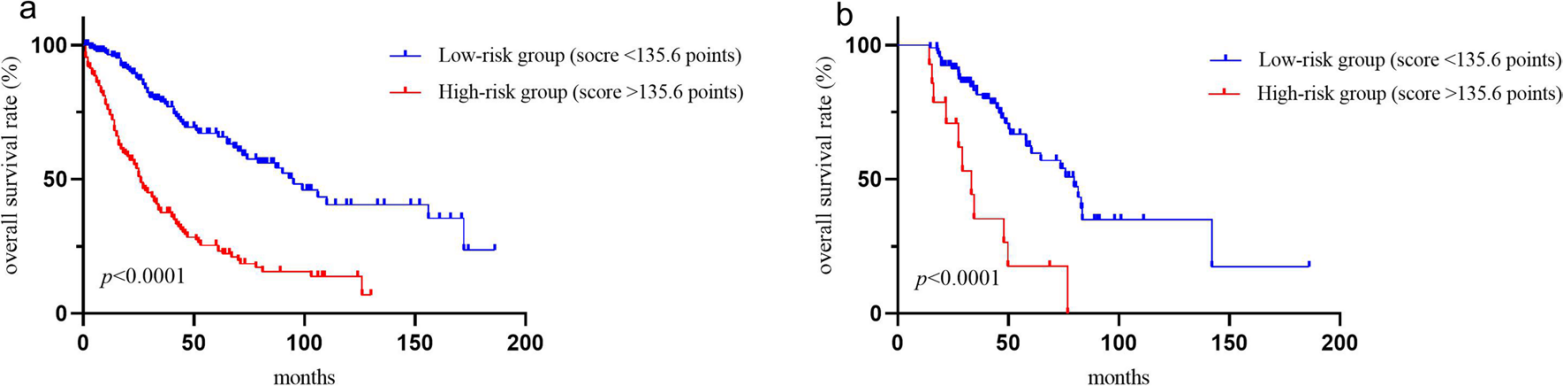
Kaplan–Meier curves of overall survival for risk stratification: training cohort (**a**) and validation cohort (**b**). The low and high score were defined according to the median score of the total population in the training cohort: < 135.6 points were defined as low-risk group and > 135.6 points were defined as high-risk group

The training cohort was stratified into two groups, namely, the high-risk group (> 135.6) and the low-risk group (< 135.6) based on the median risk score (135.6). The low-risk group exhibited significantly improved OS than the high-risk group (*p* < 0.0001). Furthermore, the Kaplan–Meier curves for the external validation cohort using the same cut-off value for OS also illustrated a distinction between the high-risk group and the low-risk group (*p* < 0.0001) (Fig. 5).

### Survival analysis

**Fig. 6:**
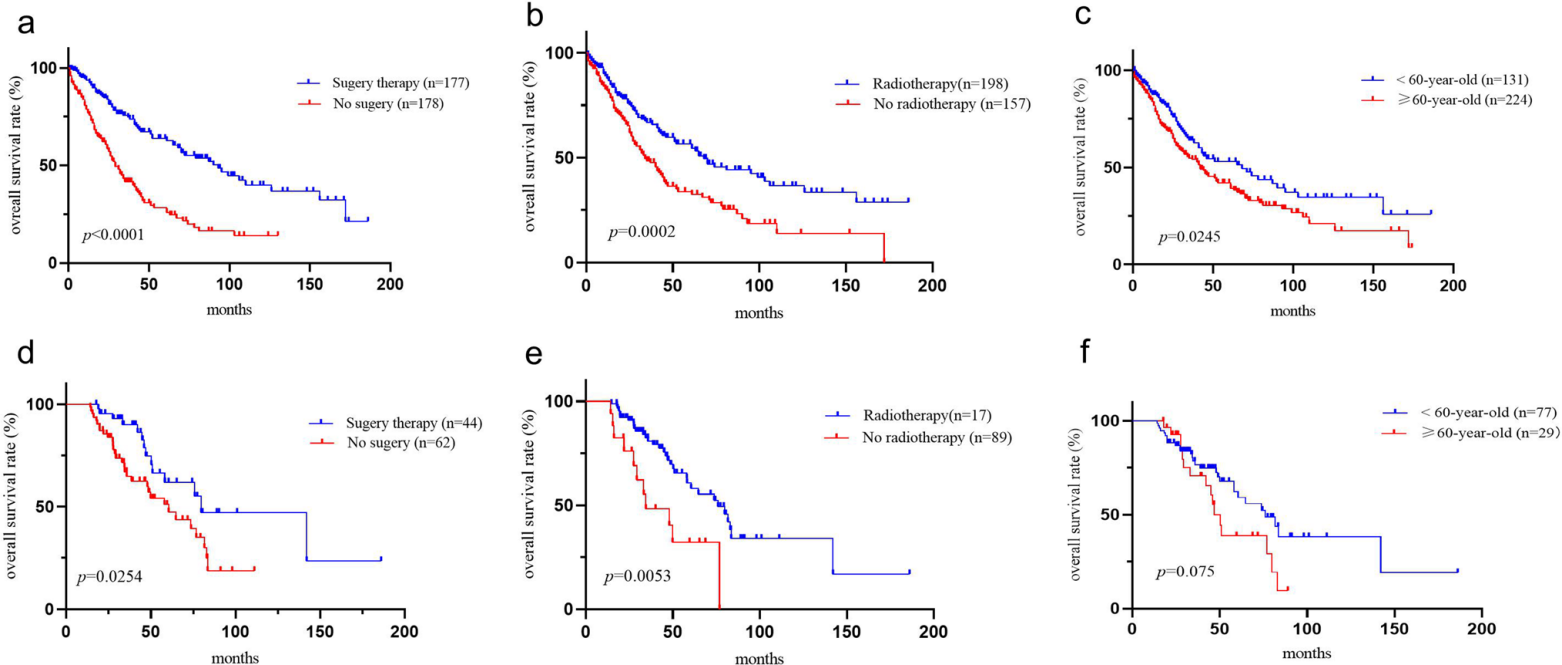
Kaplan–Meier curves for TSCC stratified by surgical treatment, radiotherapy, and age. (**a**) Survival analysis of TSCC with surgical resection in training cohort. The prognosis of thymic squamous cell carcinoma treated with surgery therapy was significantly better than those without surgery (*p* < 0.0001). (**d**) Survival analysis in external validation cohort for surgical treatment. The 5 year OS rate for TSCC patients with or without surgery treatment was 61.9% and 47.3% (*p* = 0.0254). (**b**) and (**e**) showed the subgroup analysis of radiotherapy in the training cohort and the external validation cohort, respectively. Figure (**c**) and (**f**) were age-stratified survival analyses for two cohorts, respectively. The effect of age on survival was observed in both cohort: ≥ 60 years of age was an adverse prognostic factor in patients with TSCC

The Kaplan–Meier method was employed to perform survival analysis on the training and validation cohort. The results indicated that the median OS of the training cohort and validation cohort were 46.0 months (95% CI 35.4–56.6 months) and 73.7 months (95%CI 54.6–92.8 months), respectively, as demonstrated in Additional file 1: Figure S1. The 3 year OS rates for TSCC patients were 58.2% and 71.0% in the training and validation cohorts, respectively, while the 5 year OS rates were 46.0% and 42.2%, respectively. Although longer median OS was observed in the validation cohort, the two cohorts were similar in terms of 5 year survival rates. Figure 6 illustrated the subgroup survival analysis of the training and validation cohorts.

Surgical excision demonstrated a significant disparity in outcomes across the populace (Fig. 6a, c). Patients who underwent surgical resection exhibited better outcomes than those who did not, in both the training and external validation cohorts, with p-values of < 0.0001 and 0.0254, respectively. The 5 year OS rate for patients with or without surgery treatment were 64.0% and 26.0% in the training cohort, and 61.9% and 47.3% in the validation cohort. The median OS for patients who underwent surgery and those who did not was 93.0 months (95%CI 66.0–120.0 months) and 29.0 months (95%CI 23.7–34.3 months) in the training cohort, respectively. Similarly, in the validation cohort, these values were 79.7 months (95%CI 24.5–31.7 months) and 60.5 months (95%CI 41.7–79.3 months), respectively.

As the number of patients who did not undergo surgery in the validation cohort with early-stage disease was only eight, we could not assess the effectiveness of surgery in patients with non-advanced disease and thus did not observe any significant differences in efficacy (*p* = 0.721). However, Kaplan–Meier curves for the surgical resection approach were provided in the Additional file material (Additional file 2 Figure S2). In the validation cohort, as expected, surgical resection demonstrated a statistically significant difference in outcomes in patients without distant metastases (*p* < 0.0001).

Furthermore, radiotherapy exhibited a marked difference in effectiveness in both the training and validation cohorts (Fig. 6b, e). The median OS for patients who underwent radiotherapy compared to those who did not in the training and validation cohorts were 68.0 months (95% CI 46.8–89.2 months) vs. 34.0 months (95% CI 25.2–42.8 months) (*p* < 0.001); 76.0 months (95% CI 57.6–94.4 months) vs. 34.5 months (95% CI 9.9–59.1 months) (*p* = 0.005), respectively.

## Discussion

Thymic tumors, originating from thymic epithelial cells, are solid tumors whose incidence has been increasingly reported in recent years [14]. Despite their rarity, more and more clinical and experimental studies are now dedicated to understanding this disease. Due to the limited availability of thymic tumor cell lines, the etiology of this disease is still not well understood. TSCC, which is more malignant and carries a worse prognosis than thymoma, is the most common type of thymic carcinoma, accounting for 61.8–74.8% of cases [13, 15–18]. Although the Masaoka-stage system has been widely used to determine the prognosis of TSCC, other factors are also known to impact the prognosis, rendering the system flawed.

Previous studies on the correlation between prognosis and survival in TSCC have been confined to analyzing the data from the SEER database. [12, 13]. It is vital to verify the nomogram to ensure its applicability and prevent over-fitting. Our study discovered that patients younger than 60 years, with localized SEER staging, and who received either radiation therapy or surgical treatment were associated with a significantly improved prognosis. This is the first research to validate the nomogram risk factors based on the SEER database for TSCC using external data. Table 1 provided a summary of the population characteristics of the two cohorts. The imbalance in cohorts can be attributed to the external data being from a solitary institution, which is not a multicenter database, and most of the patients in this data were diagnosed with the disease within the past 5 years, compared to the training cohort. Nonetheless, this has once again demonstrated the generalizability of our model. The study reported a 3- and 5 year overall survival rate of 58.2% and 46.0% and 71.0% and 42.2% in the SEER database and validation cohort, respectively. These results are consistent with 5 year OS rate reported in prior literature (33–65%) [5, 7, 13, 19–21].

WU J et al. [12] have observed the relationship between age and prognosis through an analysis of the SEER database, while our research has advanced this analysis by identifying a cut-off value of 60 years. However, Lim and colleague have reported that age ≥ 63 years was an independently poor prognostic factors in patients with thymic carcinomas (*p* = 0.023) [22]. It is an acknowledged fact that older individuals tend to have a greater burden of underlying comorbidities, which might impede the effectiveness of surgery and radiation therapy. Patients of different ages also have varying degrees of functional capacity, with younger patients being more capable of tolerating the hazards associated with surgical and comprehensive treatment modalities.

The Masaoka-stage system and the American Joint Committee on Cancer (AJCC) 8th Edition Tumor-Node-Metastasis (TNM) staging system are widely accepted as the standards for thymic carcinomas [1, 23]. N2 classification is given to patients with intrathoracic lymphatic metastasis, corresponding to the IVB of the TNM stage. Nevertheless, our study reveals that patients in the regional subgroup, defined as those with intrathoracic lymphatic metastasis, exhibit a superior prognosis compared to those in the distant group (defined as those with distant metastasis). Patients with only thoracic lymph node metastases may derive greater benefits from adjuvant chemotherapy and radiation therapy. SEER staging can effectively discriminate the prognosis of patients with TSCC. Therefore, it is worth considering the necessity of further improving the staging system for thymic tumors.

No study has thus far challenged the pre-eminence of surgical resection in the management of thymic tumors. Nevertheless, a considerable proportion of patients, who underwent surgical resection at an early stage, ultimately developed distant metastases and patients diagnosed at an advanced stage received limited surgical intervention in the validation cohort. Owing to the small number of early-stage patients who did not undergo surgery (n = 8), we did not perform a surgical subgroup analysis comparing early-stage versus advanced patients.

Our study underscores the importance of surgical resection in thymic tumors. Previous studies have showed that incomplete tumor resection is linked to a high recurrence rate of 65% (*p* < 0.01) [3]. In the validation cohort, we also observed that complete surgical excision was a favorable prognostic factor that improved patient survival (*p* = 0.009). However, we did not detect any significant variation in the training cohort (*p* = 0.127), which was attributed to the sample size (Additional file 3: Figure S3). Nevertheless, when we analyzed 423 cases comprehensively, a significant difference was observed between partial and complete resection. In addition, our study detected inevitable disease progression and multiple systemic metastases in some early-stage patients even after radical resection and adSuvant radiotherapy, and a lack of responsiveness to other chemotherapeutic agents in such patients. However, other patients with the same early-stage disease showed markedly different outcomes. The reasons for this are yet to be discovered.

Although tumor size did not show statistical significance in the multivariate analysis, it still warrants attention (*p* = 0.006). It cannot be disregarded that there exist some biases that hinders the survival difference of tumor size. In the research, tumors with a size of 0–5 cm were shown to be a protective factor compared to larger tumors. YE C et al. have previously shown that patients with tumor of size ≥ 7 cm experience significantly reduced survival benefits (*p* = 0.004) [24]. Further optimization of the cut-off value for tumor size in thymic carcinomas is therefore necessary.

The significance of radiotherapy in the management of thymic tumors is increasingly being acknowledged. Radiotherapy is being considered as one of the recommend therapeutic strategy to augment the survival rate of patients with thymic carcinomas of Masaoka-stage II to IVA [18, 25, 26]. The treatment modes comprise neoadjuvant radiotherapy, postoperative adjuvant and palliative radiotherapy. The unique anatomical location of thymic carcinoma poses a challenging task for radiation oncologists to ensure precise targeting of the radiation. Our study unequivocally affirms that radiotherapy is a valuable prognostic determinant for patients afflicted with TSCC.

Neither the SEER data nor the data from our institution restricted the temporal sequence of radiotherapy and surgery in the overall management of thymic tumors. Among the SEER data, it was observed that 123 (66.7%) patients received both surgery and radiotherapy, and the 5 year survival rate for this group was 65.6%. However, for the 103 (30.1%) patients who did not undergo either radiation or surgery, the 5 year survival rate was only 20.1%, which was found to be statistically significant (*p* < 0.0001).

Previous studies have demonstrated the efficacy of post-operative radiotherapy (PORT) in TCs [22, 25–28]. PORT can significantly improve disease-free and overall survival of TCs. Nevertheless, a recent study showed that the most common reasons for failure of PORT were distant metastases (32.3%), pleural metastases (22.0%), and locoregional failure (15.0%) [27]. Similarly, neoadjuvant radiotherapy has shown better benefits in thymic carcinoma [29]. However, a review suggested that the OS benefit of adjuvant chemotherapy may be confounded by the management of PORT [30]. Further exploration is still needed regarding the standardization of radiotherapy and chemotherapy modalities for the full management of TCs. Thus, high-quality prospective study data on PORT and adjuvant chemotherapy in TCs are required. It is eagerly anticipated that data from prospective studies will provide clarity on the efficacy of PORT and adjuvant chemotherapy in the management of TCs.

Regrettably, chemotherapy did not yield any significant differences in efficacy in either of the study cohorts. This outcome was attributed to the sample size. However, upon including the 356th patient in the training cohort, chemotherapy exhibited a difference in efficacy (*p* < 0.0001). Moreover, there were only seven patients did not received chemotherapy in the validation cohort. And the majority of patients, including those with early-stage and advanced disease, received chemotherapy, making it inappropriate to investigate its efficacy in the entire population. In addition, the survival benefits of chemotherapy and radiation may be confounded to some extent. The survival curve of chemotherapy for both two cohorts was depicted in Additional file 4: Figure S4.

Our study amalgamated data from our institution to verify independent predictors of TSCC. The validation cohort’s C-index value was 0.717 (95% CI 0.640–0.794). Furthermore, the AUC values for 3 and 5 years were 0.795 and 0.719, respectively. Despite the calibration curve’s suboptimal presentation concerning the conformity between the test model’s anticipated risks and the actual risks, we believe that this is attributed to the insufficient sample size.

Overall, our nomogram model provides a reasonable estimation of the actual prediction, particularly in terms of the 3 year OS rate. All the data incorporated in the nomogram are based on clinical parameters, which can help healthcare professionals tailor individual survival prognoses for their patients. Further research is encouraged to integrate additional clinical factors to enhance the model's accuracy. It is also recommended that future studies should include real-world data with more extended follow-up periods and larger sample sizes to validate the model. Moreover, since thymic tumors generally exhibit better prognosis compared to other solid tumors, longer follow-up periods, such as 8 year and 10 year survival rates, are imperative.

There are several limitations in our study that need to be addressed. Firstly, as a retrospective cohort study, there is an inherent risk of retrospective bias. Secondly, due to the unknown timing of surgery, radiotherapy and chemotherapy in the management of thymic carcinoma, as well as the lack of information on Masaoka-stage, some important factors may not have been considered in our analysis. Thirdly, the relatively small sample size, which is limited to a single institution, may have resulted in an unbalanced distribution between the cohorts, thereby affecting the accuracy and generalizability of our results.

## Conclusion

We developed a nomogram to predict 3- and 5 year survival rate for TSCC. This nomogram provides a convenient and reliable tool for assessing the condition of patients with TSCC and assisting clinicians in making decisions.

## Supplementary Information

**Additional file1: Figure S1**. Kaplan-Meier curves for overall survival in the training cohort (a) and the validation cohort (b).

**Additional file 2: Figure S2**. Kaplan-Meier curves for non-operated and surgical therapy in non-advanced patients in the training cohort (a) and the validation cohort (b).

**Additional file 3: Figure S3**. Kaplan-Meier curves for surgical resection in the training cohort (a) and the validation cohort (b).

**Additional file 4: Figure S4**. Kaplan-Meier curves for chemotherapy in the training cohort (a) and the validation cohort (b).

## Data Availability

The data sets used and/or analyzed in this study are available from the corresponding authors upon reasonable request.

## Abbreviations

TSCC: Thymic squamous cell carcinoma
SEER: Surveillance, epidemiology, and end results
TCs: Thymic carcinomas
OS: Overall survival
CI: Confidence interval
HRs: Hazard ratios
AUC: Under the curve
ICIs: Immune checkpoint inhibitors
AJCC: American joint committee on cancer
TNM: Tumor-node-metastasis
PORT: Post-operative radiotherapy
