# Supplementary figures and images for: Survival nomogram for patients with thymic squamous cell carcinoma, based on the SEER database and an external validation cohort
Source: Discov Oncol. 2023 Jun 20;14:106. doi: 10.1007/s12672-023-00720-4 (PMC10281918; doi:10.1007/s12672-023-00720-4)

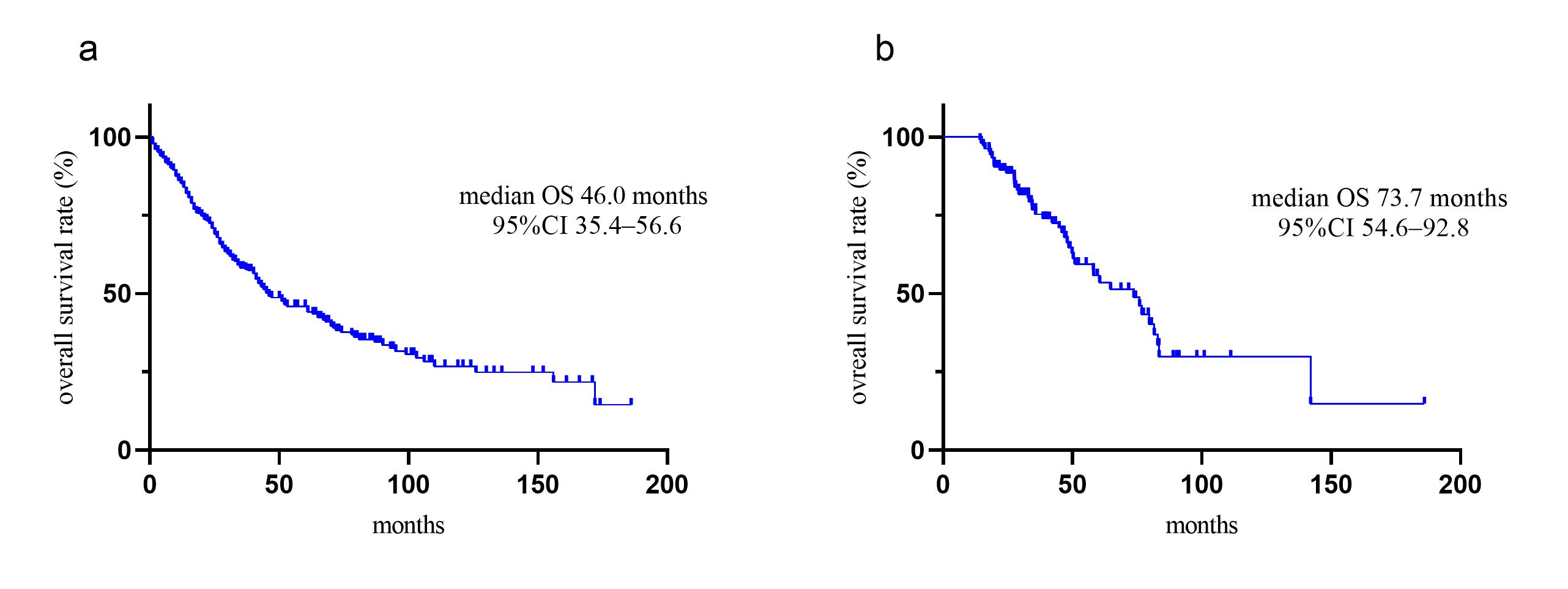

Supplement: Supplementary file 1 — Additional file1: Figure S1. Kaplan-Meier curves for overall survival in the training cohort (a) and the validation cohort (b). [file 12672_2023_720_MOESM1_ESM.jpg]

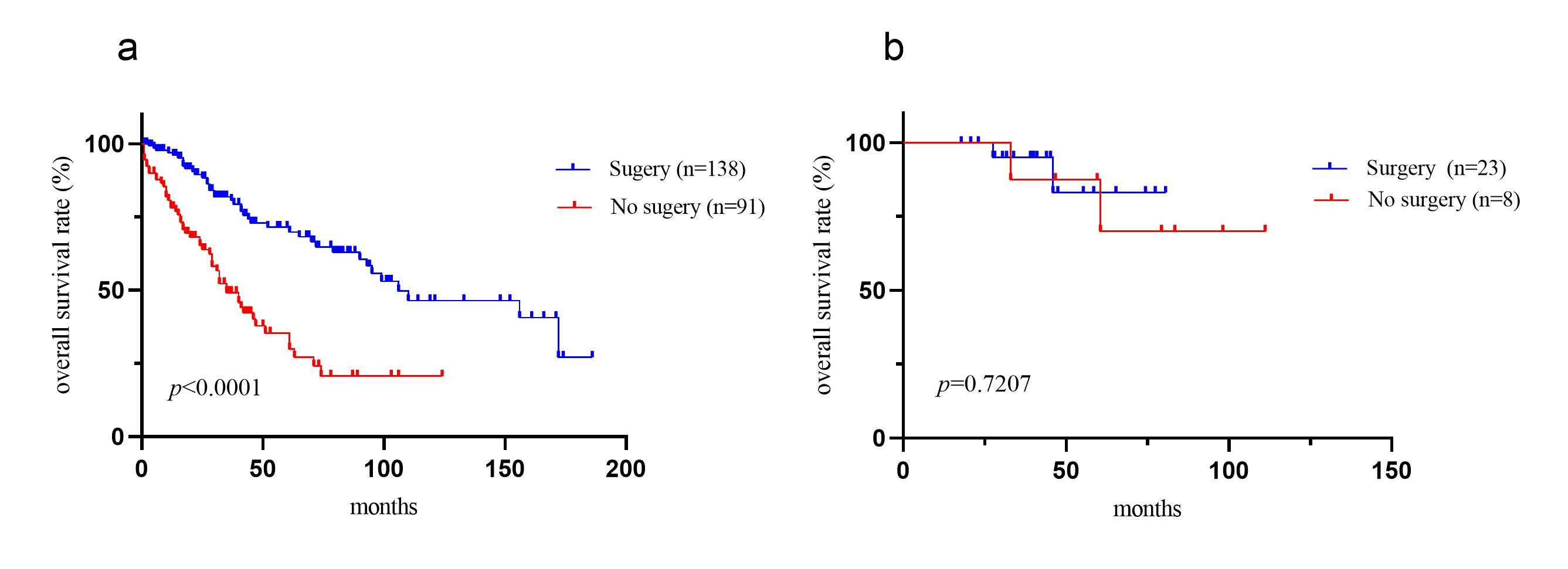

Supplement: Supplementary file 2 — Additional file 2: Figure S2. Kaplan-Meier curves for non-operated and surgical therapy in non-advanced patients in the training cohort (a) and the validation cohort (b). [file 12672_2023_720_MOESM2_ESM.jpg]

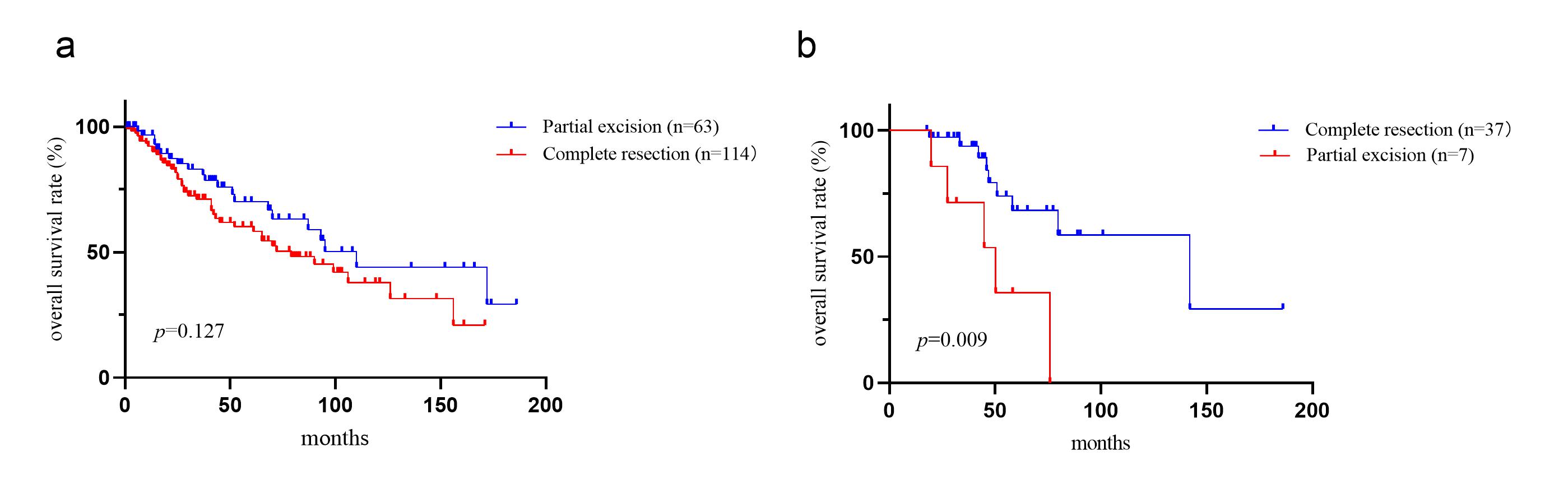

Supplement: Supplementary file 3 — Additional file 3: Figure S3. Kaplan-Meier curves for surgical resection in the training cohort (a) and the validation cohort (b). [file 12672_2023_720_MOESM3_ESM.jpg]

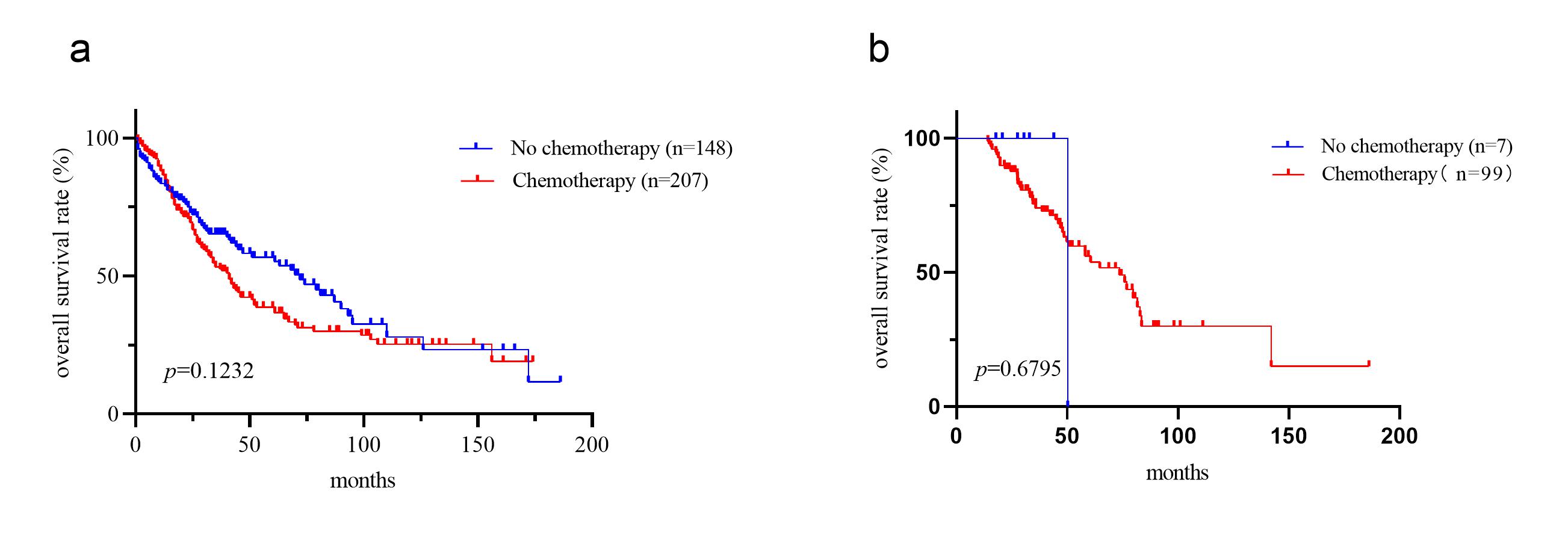

Supplement: Supplementary file 4 — Additional file 4: Figure S4. Kaplan-Meier curves for chemotherapy in the training cohort (a) and the validation cohort (b). [file 12672_2023_720_MOESM4_ESM.jpg]
